# Supplementary material for: Automatic light-adjusting electrochromic device powered by perovskite solar cell
Source: Nat Commun. 2021 Feb 12;12:1010. doi: 10.1038/s41467-021-21086-7 (PMC7881180; doi:10.1038/s41467-021-21086-7)
Supplement: Supplementary file 1 — Supplementary Information [file 41467_2021_21086_MOESM1_ESM.pdf]

Automatic light-adjusting electrochromic device powered by perovskite solar cell

Huan Ling[1], Jianchang Wu[1], Fengyu Su[1,2,3*], Yanqing Tian[1*], Yan Jun Liu[3*]

1. *Department of Materials Science and Engineering, Southern University of Science and Technology, Shenzhen, 518055, China*

2. *Academy for Advanced Interdisciplinary Studies, Southern University of Science and Technology, Shenzhen, 518055, China*

3. *Department of Electrical and Electronic Engineering, Southern University of Science and Technology, Shenzhen, 518055, China*

Supplementary Note 1

To further study the redox reactions and kinetic processes of gel ECDs, CV curves were measured at various scan rates from 20 to 400 mV/s (Supplementary Fig. 1a and Supplementary Fig. 1b). The dimerization of viologen radicals induced by spin-paring emerged with the increase of the alkynyl substituents attached to the nitrogen atom of viologen. With the increase of scan rates, the cathodic peak approached to the negative direction and the anodic peak moved toward to the opposite sites for both two viologens. Remarkably, a new oxidation peak emerged gradually for MPV as shown in Supplementary Fig.1b. While, this phenomenon was not observed for DPV. This difference could be explained by the chemical equilibrium as written in Eq (S1). When the MPV-gel ECD was scanned at a slow rate, the MPV^{+} molecules involved in the reduction process were almost converted to the $MPV^{\bullet}$. At the same time, the alkynyl

group served as the electron-donor group to stabilize the radical cations through the p-π interactions in two vertical directions. As a result, solo oxidation peak of MPV^{+}/ $MPV^{\bullet}$ was observed in the CV curves.

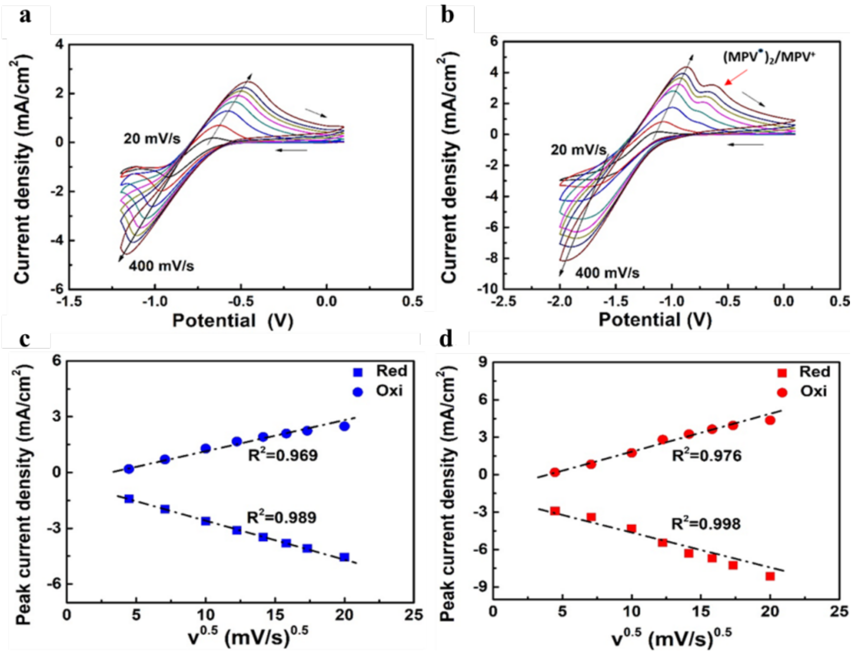

Supplementary Fig. 1. **Kinetic studies of DPV and MPV based ECDs**. CV curves under different applied scan rates at 20, 50, 100, 150, 200, 250, 300, and 400 mV/s ; and corresponding linear plots of peak current densities versus roots of scan rates for ECDs based on DPV (a, c) and MPV (b, d).

$$MPV^{\bullet} \rightleftharpoons (MPV^{\bullet})_2 \qquad \text{equation (S1)}$$

However, in the same time interval, larger quantities of $MPV^{\bullet}$ molecules generated and failed to escape from cathodic electrode at a high scan rate. The diffusion process failed to carry on completely and the effect of p-π interactions was limited, which induced the chemical equilibrium (Equation S1) to the right. As a result, the

oxidation peak from the radical dimer $(MPV^{\bullet})_2$ to MPV^{+} appeared. Nevertheless, when two alkynyl groups were introduced in the same viologen moiety, simultaneous intermolecular interactions occurred for two alkynyl groups in same DPV molecule contributed to “lock” the molecule itself to reduce the probability of direct contact of intermolecular bipyridine moieties, which basically reduced the aggregation of radical cation species. In addition, two sets of vertical π bonds provides intermolecular interactions to stabilize the produced free radicals in different directions. Consequently, the oxidation peak of radical dimer wasn’t observed for DPV-based gel ECD. Supplementary Fig. 2 demonstrated several interactions between radical cation species and alkynyl groups. This assumption was further confirmed by the following studies on diffusion processes.

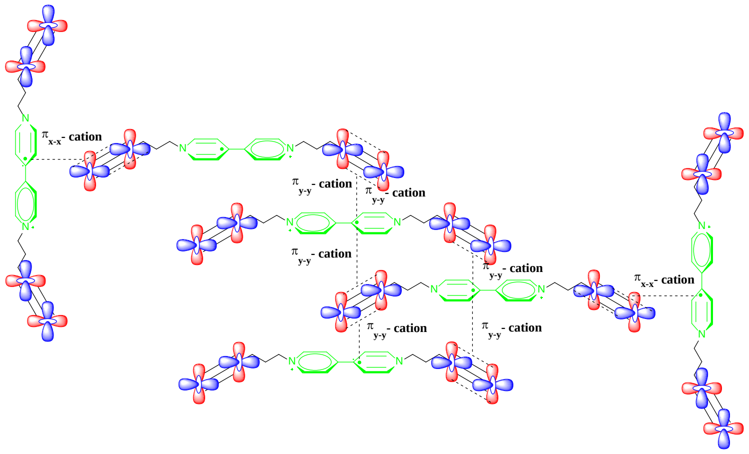

Supplementary Fig. 2. Proposed interactions between alkynyl groups and radical cations of bipyridine.

As plotted in Supplementary Fig. 1c and Fig. 1d, linear relationships of current densities of anodic and cathodic peaks versus scan rates and the roots of scan rates were observed. The high linearity with R^2 larger than 0.96 indicated that the first oxidation/reduction of DPV^{2+} and MPV^{+} were limited by diffusion-controlled process.

Generally, the diffusivities (*D*) in typical one electron exchange process can be calculated by Randles−Sevcik equation[51]. The equation at r.t. (298K) was given in Equation (S2):

$$i_p = 269000\ n^{1.5} A D^{0.5} C_o v^{0.5} \qquad \text{equation (S2)}$$

where the peak current density ip was obtained at the cathodic peak, the number of involved electron (n) is 1, the active area (A) is 2 cm^2, the original concentration is 0.06 M. The diffusivities (D) can be calculated by the slope of i_p versus $v^{0.5}$, which are 4.75 $\times 10^{-11}$ and 1.45 $\times 10^{-10}$ cm^2/s for DPV and MPV-based gel ECDs, respectively. The lower D of DPV than MPV could be attributed to the larger molecule size of former. In addition, the larger D of MPV suggested that the MPV cost less time to diffuse to the cathodic electrode from gel bulk, along with the higher reduction potential, which led to higher current density than that of DPV.

Supplementary Note 2

Annual energy consumption estimation

The power to drive TEV based ECD to the deep colored state was 19.1 mJ/cm^2 according to Fig 2e. If the smart window works 8 cycles per day, the annual energy consumption was calculated about 55.8 J/cm^2 (0.16 kW·h/m^2) per year approximately.

The power to sustain the deep colored state of DPV was 4.4 mW/cm^2 according to Fig 2f. If the smart window works 8 h per day, the annual energy consumption was calculated 4.6*10^4 J/cm^2 (128.4 kW·h/m^2) per year approximately.

The output power of PSC was approximately 18 mW/cm^2 according to Supplementary Fig. 6. If the PSC works 8 h per day, the annual harvested energy was about 2.1 *10^5 J/cm^2 (525.6 kW·h/m^2).

Considering the power change of ECD in the whole day under a variable light intensity, the actual annual energy consumption or harvesting would be lower than estimated values for both ECD and PSC.

a **b**

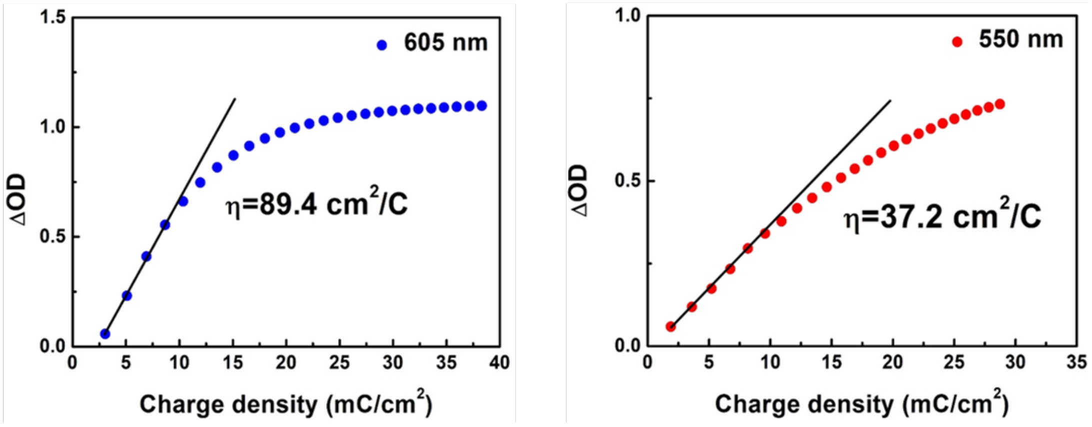

Supplementary Fig. 3. **Calculation of Coloration efficiency of ECDs**. Optical density versus charge density of ECD based on DPV (a) and MPV (b).

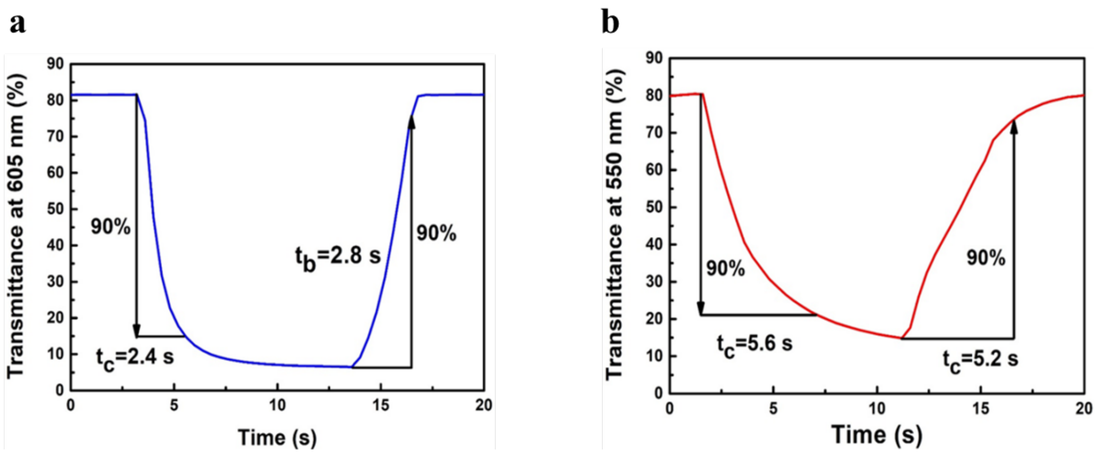

Supplementary Fig. 4. **Characterization of response time of ECDs**. Optical transmittance change with time of ECD based on DPV (a) and MPV (b) in a cycle between the switching voltages of 1.6 V and -0.3 V.

a b

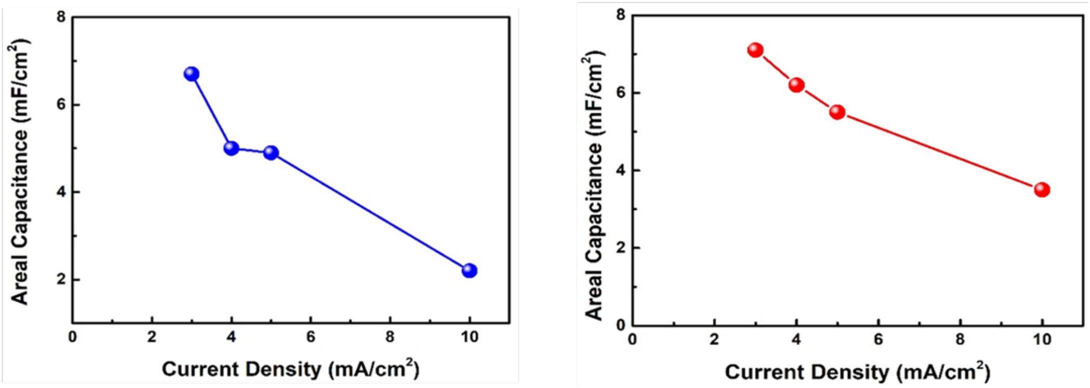

Supplementary Fig. 5. **Areal capacitances of ECDs**. Corresponding areal capacitance for ECD based on DPV (a) and MPV (b) at specific current densities.

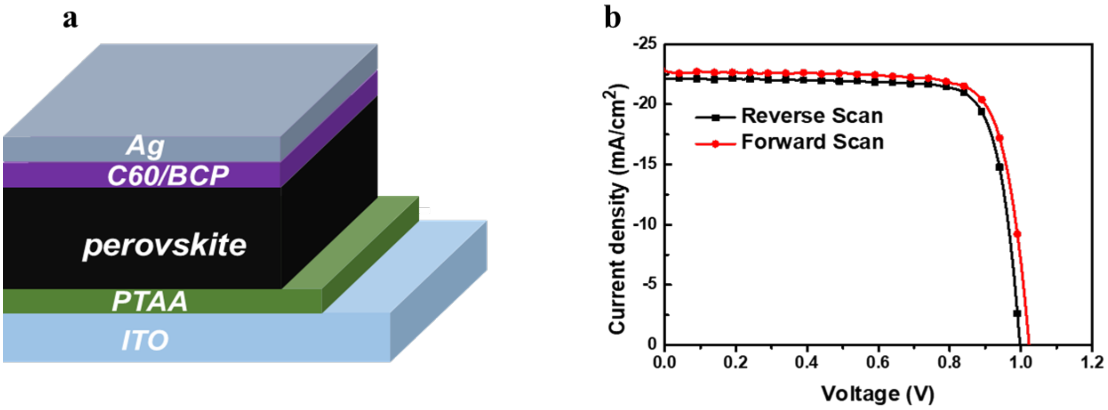

Supplementary Fig. 6. **Structure and characterization of PSCs**. a. The configuration of fabricated PSCs; b. Photocurrent density-voltage (*J*-*V*) curves of PSCs.

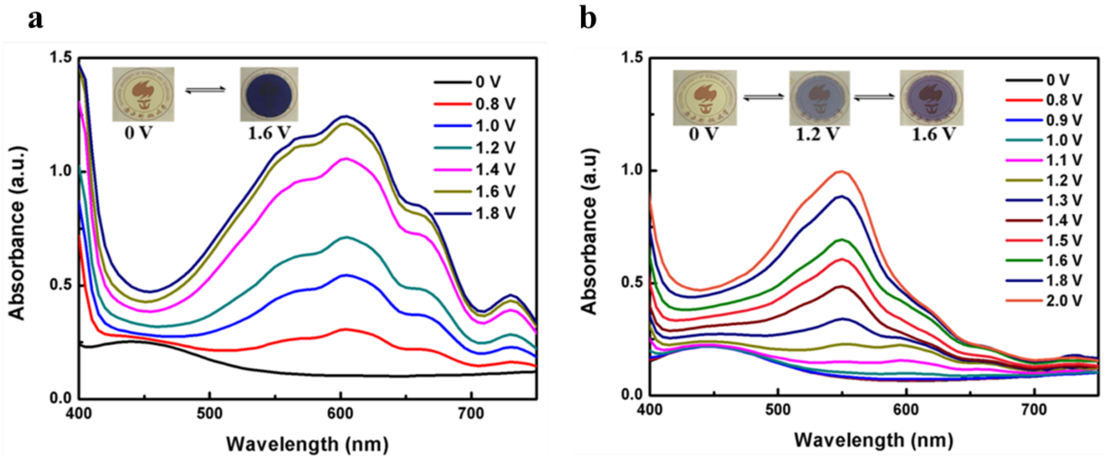

Supplementary Fig. 7. **The spectroelectrochemical characterization of ECDs**.

UV-*vis* absorption spectra and photographs of ECDs based on DPV (a) and MPV (b) under different voltages.

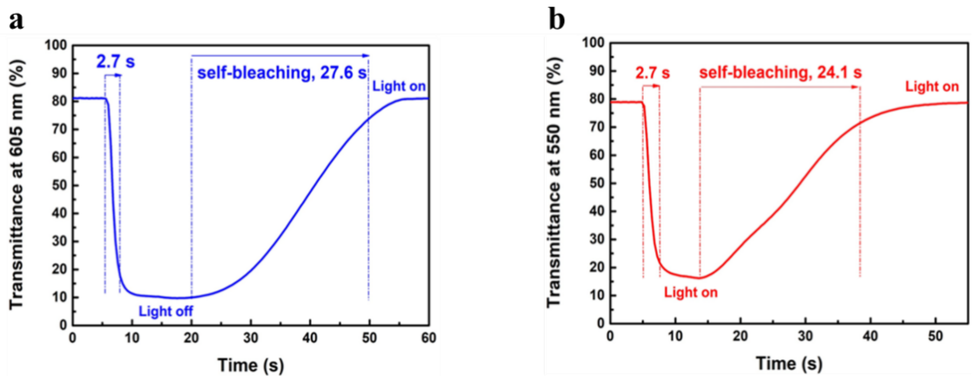

Supplementary Fig. 8. **Characterization of response time of PSCs-powered ECDs.** Transmittance change with time of PSC-powered ECDs (a: DPV, b: MPV) under strong and no light at maximum absorption peak.

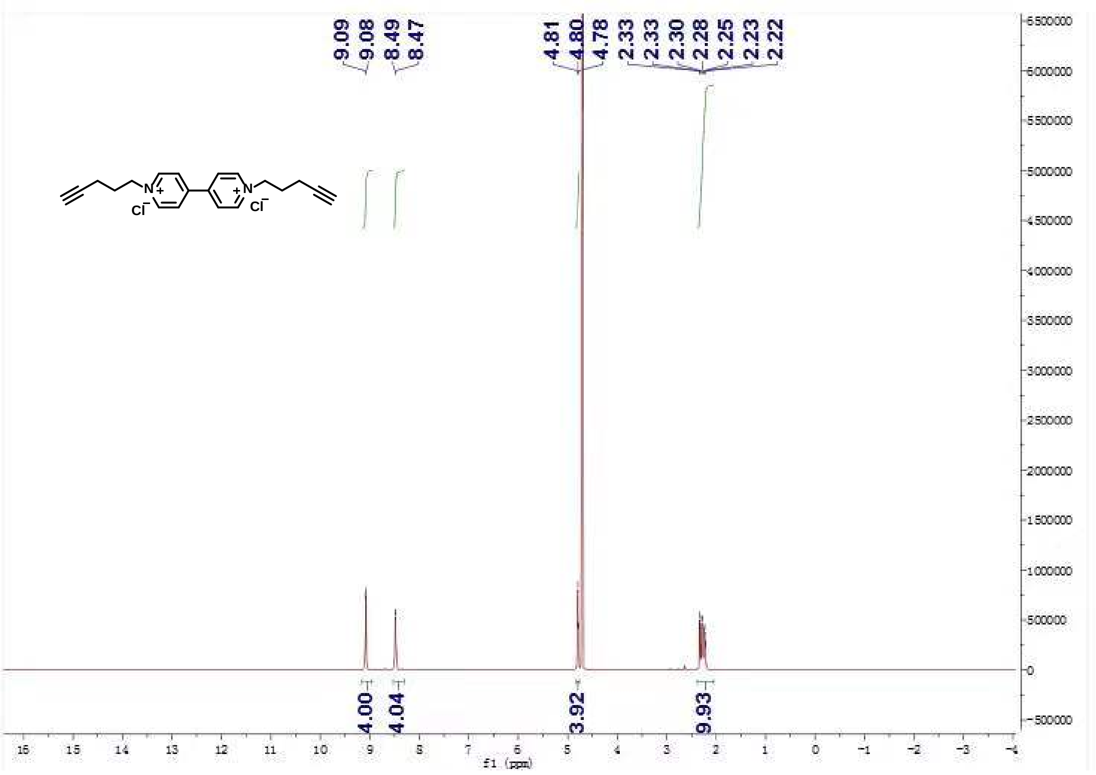

Supplementary Fig. 9. ^{1}H NMR spectrum of DPV.

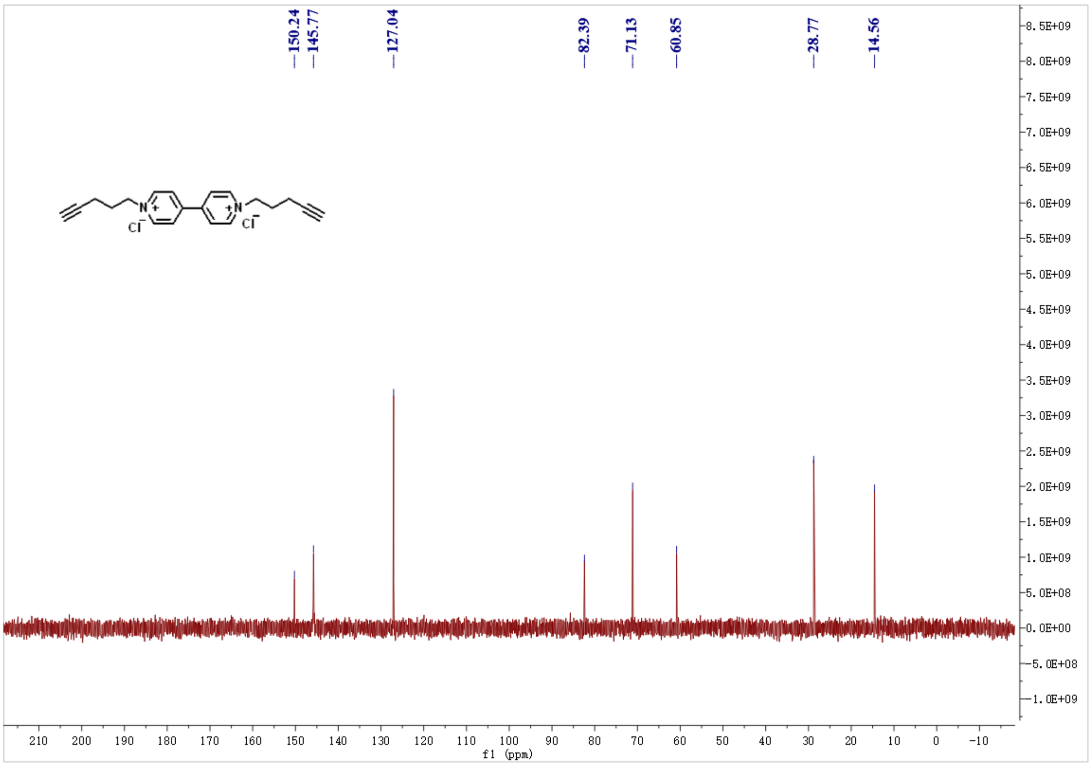

Supplementary Fig. 10. ^{13}C NMR spectrum of DPV.

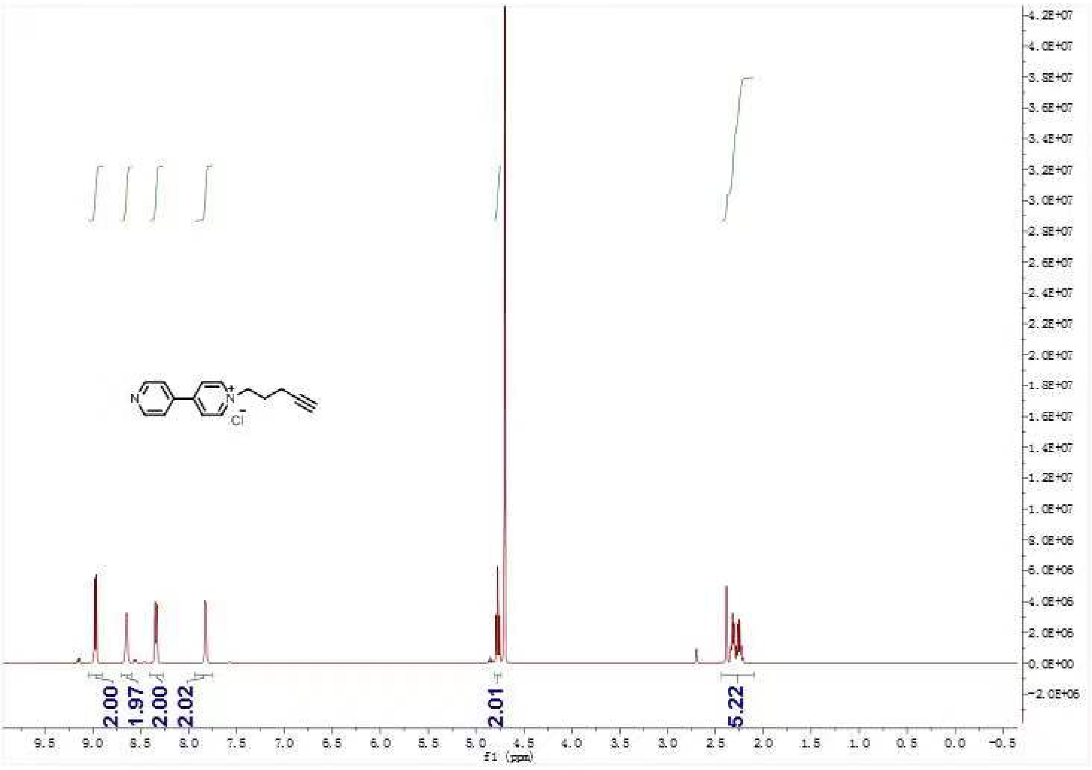

Supplementary Fig. 11. ^{1}H NMR spectrum of MPV.

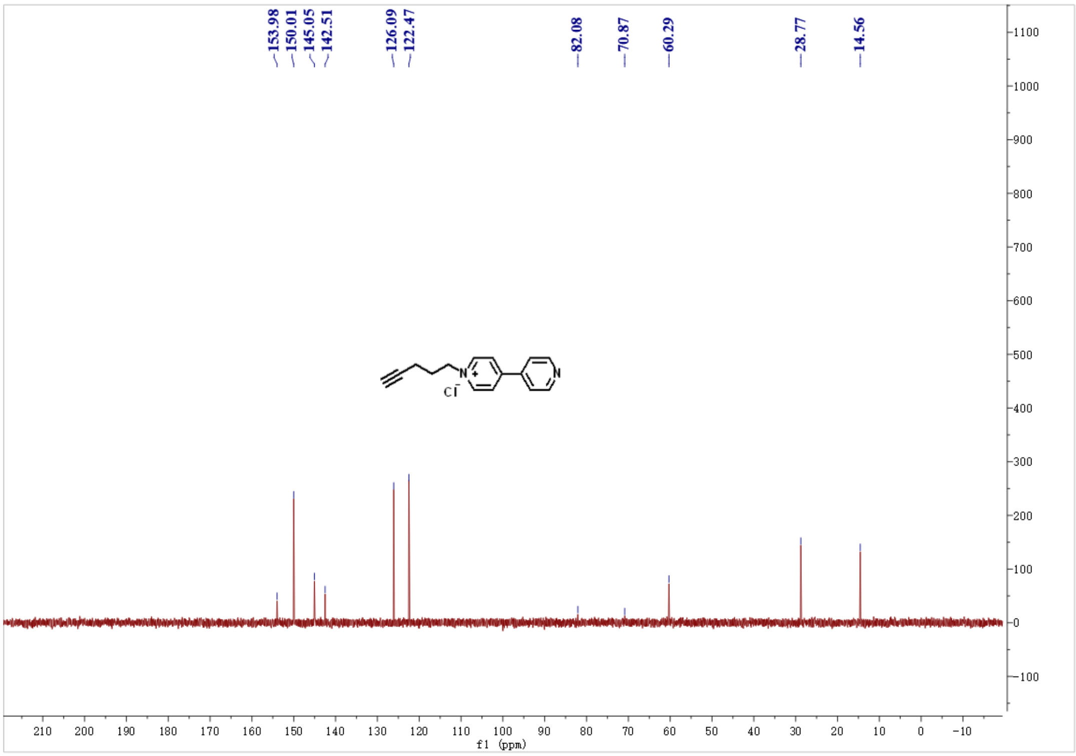

Supplementary Fig. 12. ^{13}C NMR spectrum of MPV.

Positive mode

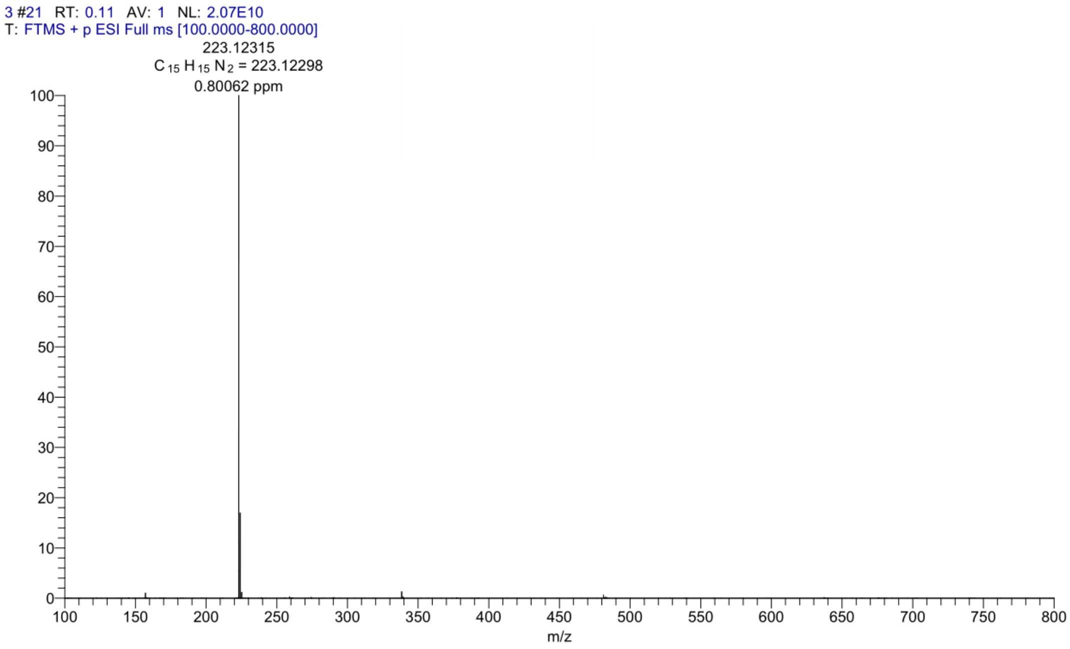

Supplementary Fig. 13. HRMS spectrum of MPV.

Supplementary Table 1. Performances of some viologen-based ECDs

EC materials	ΔT(%)	Stability	Ref
Monoheptyl-viologen	>80	3600 s without degradation (72 Cycs)	1
Diheptyl-viologen	>80	3600 s without degradation (~67 Cycs)	1
Vinyl benzyl viologen	65	60.5% remained after 10000 Cycs	2
DTFMBzV[a]	63.5	61.6% remained after 10000 Cycs	3
Nonyl viologen	55.2	53.8% remained after 10000 Cycs	4
PBT[b]	60	About 42% remained after 60000 Cycs	5
CPD[c]	63	53.7% remained after 40000 Cycs	6
DPV(this work)	74.3	60.9% remained after 70000 Cycs	-

Cycs: cycles; *a*: 1,1′-bis(3,5-bis(trifluoromethyl)-benzyl)-4,4′-bipyridine-1,1′-diium, *b*: 1,4-bis[((N-phosphono-2-ethyl)-4,4-bipyridinium)-methyl]-benzene tetrachloride, *c*: 1-(9-hexyl-9H-carbazole)-1-(propylphosphonicacid)-4,4-bipyridilium dichloride

Supplementary References

1. Kim JW, Myoung JM. Flexible and Transparent Electrochromic Displays with Simultaneously Implementable Subpixelated Ion Gel-Based Viologens by Multiple Patterning. *Adv. Funct. Mater.* **29**, 1808911 (2019).
2. Kao SY, Lu HC, Kung CW, Chen HW, Chang TH, Ho KC. Thermally Cured Dual Functional Viologen-Based All-in-One Electrochromic Devices with Panchromatic Modulation. *ACS Appl. Mater. Interfaces* **8**, 4175-4184 (2016).
3. Yu H-F, Chen K-I, Yeh M-H, Ho K-C. Effect of trifluoromethyl substituents in benzyl-based viologen on the electrochromic performance: Optical contrast and stability. *Sol. Energy Mater. Sol. Cells* **200**, 110020 (2019).
4. Lu HC, Kao SY, Yu HF, Chang TH, Kung CW, Ho KC. Achieving Low-Energy Driven Viologens-Based Electrochromic Devices Utilizing Polymeric Ionic Liquids. *ACS Appl. Mater. Interfaces* **8**, 30351-30361 (2016).
5. Weng D, Shi Y, Zheng J, Xu C. High performance black-to-transmissive electrochromic device with panchromatic absorption based on TiO2-supported viologen and triphenylamine derivatives. *Org. Electron.* **34**, 139-145 (2016).
6. Li M, Wei Y, Zheng J, Zhu D, Xu C. Highly contrasted and stable electrochromic device based on well-matched viologen and triphenylamine. *Org. Electron.* **15**, 428-434 (2014).
