# Supplementary material for: Automatic light-adjusting electrochromic device powered by perovskite solar cell
Source: Nat Commun. 2021 Feb 12;12:1010. doi: 10.1038/s41467-021-21086-7 (PMC7881180; doi:10.1038/s41467-021-21086-7)
Supplement: Supplementary file 3 — Solar Cells Reporting Summary [file 41467_2021_21086_MOESM3_ESM.pdf]

nature research

Corresponding author(s): Fengyu Su, Yanqing Tian, Yan Jun Liu

Solar Cells Reporting Summary

Nature Research wishes to improve the reproducibility of the work that we publish. This form is intended for publication with all accepted papers reporting the characterization of photovoltaic devices and provides structure for consistency and transparency in reporting. Some list items might not apply to an individual manuscript, but all fields must be completed for clarity.

For further information on Nature Research policies, including our data availability policy, see Authors & Referees.

Experimental design

Please check: are the following details reported in the manuscript?

1. Dimensions

Item	Yes	No	Details
Area of the tested solar cells	☒	☐	Fabrication of perovskite solar cells in Method part
Method used to determine the device area	☒	☐	Fabrication of perovskite solar cells in Method part

2. Current-voltage characterization

Item	Yes	No	Details
Current density-voltage (J-V) plots in both forward and backward direction	☒	☐	Fig S6 in supporting information
Voltage scan conditions<br>*For instance: scan direction, speed, dwell times*	☒	☐	Fabrication of perovskite solar cells in Method part
Test environment<br>*For instance: characterization temperature, in air or in glove box*	☒	☐	Fabrication of perovskite solar cells in Method part
Protocol for preconditioning of the device before its characterization	☒	☐	Fabrication of perovskite solar cells in Method part
Stability of the J-V characteristic<br>*Verified with time evolution of the maximum power point or with the photocurrent at maximum power point; see ref. 7 for details.*	☐	☒	This stability was not tested

3. Hysteresis or any other unusual behaviour

Item	Yes	No	Details
Description of the unusual behaviour observed during the characterization	☐	☒	No unusual behavior was observed
Related experimental data	☐	☒	No

4. Efficiency

Item	Yes	No	Details
External quantum efficiency (EQE) or incident photons to current efficiency (IPCE)	☐	☒	EQE is not necessary for our work
A comparison between the integrated response under the standard reference spectrum and the response measure under the simulator	☐	☒	EQE is not necessary for our work
For tandem solar cells, the bias illumination and bias voltage used for each subcell	☐	☒	Our cells were only fabricated for single solar cells

5. Calibration

Item	Yes	No	Details
Light source and reference cell or sensor used for the characterization	☒	☐	Fabrication of perovskite solar cells in Method part
Confirmation that the reference cell was calibrated and certified	☒	☐	Fabrication of perovskite solar cells in Method part

Calculation of spectral mismatch between the reference cell and the devices under test

- [ ] Yes
- [x] No

The light spectrum used in measurement matched with the reference silicon cell, and we have not calculated the spectral mismatch between the reference cell and the tested devices

6. Mask/aperture

Size of the mask/aperture used during testing

- [x] Yes
- [ ] No

Fabrication of perovskite solar cells in Method part

Variation of the measured short-circuit current density with the mask/aperture area

- [x] Yes
- [ ] No

There is no obvious change in the measured short-circuit current density with or without masks

7. Performance certification

Identity of the independent certification laboratory that confirmed the photovoltaic performance

- [ ] Yes
- [x] No

This is a common PCE and does not require certification

A copy of any certificate(s)
Provide in Supplementary Information

- [ ] Yes
- [x] No

No

8. Statistics

Number of solar cells tested

- [ ] Yes
- [x] No

The device with very similar structure has been reported in our previous work ；Nat. Energy 5, 131–140 (2020)

Statistical analysis of the device performance

- [ ] Yes
- [x] No

The device with very similar structure has been reported in our previous work ；Nat. Energy 5, 131–140 (2020)

9. Long-term stability analysis

Type of analysis, bias conditions and environmental conditions
For instance: illumination type, temperature, atmosphere humidity, encapsulation method, preconditioning temperature

- [ ] Yes
- [x] No

The device with very similar structure has been reported in our previous work；Nat. Energy 5, 131–140 (2020)
